# Age, pathogen exposure, but not maternal care shape offspring immunity in an insect with facultative family life

**DOI:** 10.1186/s12862-017-0926-y

**Published:** 2017-03-07

**Authors:** Fanny Vogelweith, Maximilian Körner, Susanne Foitzik, Joël Meunier

**Affiliations:** 1 Zoological Institute, Evolutionary Biology, Johannes-Gutenberg University of Mainz, Mainz, Germany; 2 Institut de Recherche sur la Biologie de l’Insecte, UMR CNRS 7261, François-Rabelais University of Tours, Tours, France

**Keywords:** Developmental stage, Instar, Family life, *Forficula auricularia*, Insect immunity, *Metarhiziumbrunneum*, Trade-off

## Abstract

**Background:**

To optimize their resistance against pathogen infection, individuals are expected to find the right balance between investing into the immune system and other life history traits. In vertebrates, several factors were shown to critically affect the direction of this balance, such as the developmental stage of an individual, its current risk of infection and/or its access to external help such as parental care. However, the independent and/or interactive effects of these factors on immunity remain poorly studied in insects.

**Results:**

Here, we manipulated maternal presence and pathogen exposure in families of the European earwig *Forficula auricularia* to measure whether and how the survival rate and investment into two key immune parameters changed during offspring development. The pathogen was the entomopathogenic fungus *Metarhizium* *brunneum* and the immune parameters were hemocyte concentration and phenol/pro-phenoloxidase enzyme activity (total-PO). Our results surprisingly showed that maternal presence had no effect on offspring immunity, but reduced offspring survival. Pathogen exposure also lowered the survival of offspring during their early development. The concentration of hemocytes and the total-PO activity increased during development, to be eventually higher in adult females compared to adult males. Finally, pathogen exposure overall increased the concentration of hemocytes—but not the total-PO activity—in adults, while it had no effect on these measures in offspring.

**Conclusions:**

Our results show that, independent of their infection risk and developmental stage, maternal presence does not shape immune defense in young earwigs. This reveals that pathogen pressure is not a universal evolutionary driver of the emergence and maintenance of post-hatching maternal care in insects.

## Background

Most living organisms are parasites [1]. By altering the growth, fecundity, and survival of their hosts, they represent a strong selective force that drives the evolution of multiple defense in their hosts [2]. To limit the costs of pathogen infections, hosts typically depend on their immune system [2]. In insects, an important part of this defense relies on the coordinate action of non-specific and constitutive mechanisms, among which textbook examples involve hemocytes and phenoloxidase [3]. Hemocytes are immune cells that circulate in the hemolymph and are involved in recognition and encapsulation of pathogens [4]. Conversely, phenoloxidase mostly mediates the melanization of foreign objects and operates through the activation of the prophenoloxidase cascade, its inactive precursor typically stored in the hemolymph and the hemocytes [5].

Investing into immunity is costly and individuals are thus expected to adjust this investment to their current risk of infection, their general condition and/or their potential access to external help provided by group members [6]. Many vertebrates and invertebrates were shown to prophylactically increase their investment into immunity when the risk of infection is high, e.g. due to the presence of pathogens or of possibly infected individuals in their vicinity [7–9]. For example, populations of the small ground finch *Geospiza fuliginosa* that lived on islands with a high parasite prevalence invested more in their immune system compared to birds under low parasite pressure [10]. Conversely, individuals from the same species/population experiencing favorable conditions either during development and/or adult life are also often able to invest more into energetically costly traits, such as immune defenses [11, 12]. In line with this prediction, large and/or well-nourished individuals are typically known to exhibit higher concentrations of immune components in their blood or hemolymph than small and light ones at the population level [12, 13]. Finally, how much an individual invests into its immunity may also depend on the help it has received or will receive from others, i.e. on the expression of social immunity [14, 15]. Social immunity is a well-studied phenomenon in eusocial insects, where it can take multiple forms such as allo-grooming and hygienic behaviors [14, 16], but is also known to play a central role in simple family units via parental care. The effect of parental care on offspring immunity is well documented in vertebrates, with examples showing that post-hatching parental care enhances the immune response of young barn swallows *Hirundo rustica* [17] or that parental deprivation reduces the immunocompetence of juveniles in mice [18] and rats [19]. Comparatively, the effects of post-hatching parental care on offspring immunity are less clear in invertebrates, with only one study showing that parental deprivation reduces the lytic activity of larval exudate—a mediator of social immunity—in the burying beetle *Nicrophorus vespilloides* [20].

Interestingly, the influence of parental care on offspring immunity may depend on the age of the offspring and their risks of pathogen infection. In many vertebrates and invertebrates, immunocompetence increases during development [21–24]. Consequently, the effects of parental care on offspring immunity could be limited to the early stages of development (when parents interact with their juveniles) and then disappear when these juveniles have developed their own immune defenses. On the other hand, parental care facilitates offspring development with effects often reaching into adulthood, so that immune defenses could still be altered long after parents stopped caring for their offspring. Finally, the risk of infection could also determine how much parents invest into the care of their juveniles [25] and thus how much the offspring can invest into their own immune defense. For instance, the presence of pathogens in the environment has been shown to increase the expression of parental care in the frog *Hylophorbus rufescens*, as well as in humans, which in turn results in higher survival rates of pathogen-exposed offspring [26, 27].

In this study, we investigated the simultaneous and interactive effects of early maternal presence, early exposure to pathogens and developmental stage on offspring immunity in the European earwig *Forficula auricularia*. In this insect, females provide multiple forms of care to their juveniles (called nymphs) during 2 weeks following egg hatching, such as food provisioning, allo-grooming and protection against predators [28]. Nevertheless, earwig nymphs are mobile, can forage on their own and are thus typically capable to develop and survive in the absence of a tending mother [28, 29]. Here, we conducted a 2x2 full-factorial experiment in which we manipulated the presence or absence of a mother, as well as the presence or absence of the entomopathogenic fungus *Metarhizium* *brunneum* in the nest during the two first weeks post egg hatching (i.e. the period of family life). We then measured nymph survival and immune defenses at the 2nd, 3rd and 4th developmental instars, as well as in the adults. Overall, we expect that maternal presence improves the short- and long-term survival of offspring reared in a previously contaminated nest. In terms of immunity, we predict that this positive effect of maternal presence translates in either a maternally-driven increase in the offspring’s capability to invest into personal immune defenses (for instance due to the early accumulation of maternally-provided resources) or a maternally-driven decrease of offspring’ investment into personal immunity (the immune protection ensured by maternal care could allow nymphs to shift their investment from immunity to other important traits such as growth). Finally, if maternal care has limited or no effect on offspring immunity, we predict offspring immune defenses to increase with age and with early pathogen exposure, but these effects to be independent of early maternal presence.

## Methods

### Insects rearing

Adult *F. auricularia* earwigs were caught in July-August 2015 in Mainz, Germany (49°58'20.5"N 8°11'42.3"E). Immediately after field sampling, earwigs were distributed among plastic containers (37 × 22 × 25 cm) grounded with humid sand. These adults were then allowed to mate freely for 4 months. Thereafter, all females were removed from their containers to mimic dispersal, a behavior they typically express under natural condition prior to egg laying [30]. The females were isolated in Petri dishes (9 cm diameter) that were furnished with moist sand, maintained under winter conditions (15°C in darkness) and provided with a diet of *ad libitum* standard food (food composition detailed in [31]). Each Petri dish was then checked twice a week for eggs. Food provisioning was stopped when eggs were found, as females typically cease to feed between egg laying and hatching [28]. At egg hatching, all clutches were transferred to and maintained under summer conditions (18-20°C D:L) until the end of the experiment (conditions detailed in [32]).

### Experimental design

A total of 98 clutches were used to measure the effects of early maternal presence and/or early pathogen exposure on two immune parameters on nymphs and young adults. Each clutch was culled to 35 nymphs 1 day after hatching (i.e. 1st instar nymphs) and then transferred to Petri dishes either with (1) their own mother and contaminated sand (*n* = 25), (2) their own mother and non-contaminated sand (*n* = 24), (3) no mother and contaminated sand (*n* = 25) or (4) no mother and non-contaminated sand (*n* = 24). The contaminated and non-contaminated sands were created by preliminary grounding each recipient Petri dish (9 cm diameter) with humid sand and then sprinkling the sand with either 100 μl of a conidiospore solution of *M. brunneum* diluted in 0.05% Tween (10^7^ spores/ml) or with 100 μl of a control spore-free solution of 0.05% Tween, respectively. *M. brunneum* is a common entomophagous fungus in the soil, which is known to infect and reduce the survival of a wide range of insects (including earwigs) in nature, but against which the roles of hemocyte concentration and phenoloxidase activity remain largely unclear [33–35]. On day 14 after egg hatching, all tending mothers were removed from their group of nymphs (when applicable) to mimic natural family dispersal [29]. Six days later, each group of nymphs was transferred to a large Petri dish (14 cm diameter) grounded with non-contaminated sand and maintained as such until they reach adulthood. Note that adult males and females produced in each family were separated at emergence to ensure virginity and avoid inbreeding at the time of immune measurements (see below) [36]. All animals were provided with an *ad libitum* amount of standard food changed twice a week (detailed food composition in [31]).

We followed offspring survival during their development by counting all group members either five (2nd, 3rd and 4th developmental instar) or ten (adults) days after the first individual of each clutch molted into the next developmental instar. Note that 1st instar nymphs molt into their 2nd instar approximately 12 days after egg hatching [32, 37]. The days five and ten were chosen to ensure that (almost) all group members reached the new instar (or adulthood) on the day of counting (see details on developmental times in [38]). After counting, we randomly sampled two nymphs per developmental instar (and one adult male and one adult female per group), weighed these individuals to the nearest 0.001 mg using a microscale (model MYA5; PESCALE, Bisingen, Germany), and used them for immune measurements (see below). Note that these animals were subtracted for the calculation of survival rates.

### Measurement of the two immune parameters

We measured two key immune parameters in 2nd, 3rd and 4th instar nymphs, as well as in adult males and females: the total-PO activity and the concentration of circulating hemocytes. Total-PO activity was defined as the sum of phenoloxidase (PO) and prophenoloxidase (PPO) activities, therefore reflecting the immunocompetence of an individual in terms of both already activated and not-yet activated phenoloxidase enzymatic cascade. Note that earwig individuals cannot be sexed until they reach adulthood. In each of the two nymphs sampled per instar, between 0.2 to 0.5 μl of hemolymph was first extracted with a glass capillary, while 1μl was extracted in each adult male and female (see above). These extracts were immediately diluted in 11 μl (for nymphs) or 25 μl (for adults) of cold sodium cacodylate/CaCl_2_ buffer (0.01 M sodium cacodylate, 0.005 M CaCl2; pH 6.5) to measure the two immune parameters.

The concentration of all hemocytes (i.e. independent of their type and thus of their specific immune function) was measured immediately after hemolymph extraction, using 10 μl of diluted hemolymph of the nymph and 10 μl of the diluted hemolymph of each male and female. This counting was done using a Neubauer Improved Haemocytometer and a microscope (magnification x 400), as described in [39].

Total-PO activity was spectrophotometrically measured using a standard protocol described in [39]. Specifically, the diluted hemolymph of one nymph (volume = 1.5 μl) and 16 μl remaining of the diluted hemolymph of each male and female were frozen at -30°C to optimize the measurement of total-PO activity. Each sample of frozen hemolymph was then thawed on ice and centrifuged for 5 min at 4°C (4000 × g). Five μl of the resulting supernatant was then added to a microplate well containing 20 μl of PBS and 140 μl of chymotrypsin solution (Sigma C-7762, 0.07 mg/ml of distilled water). A volume of 20 μl of L-dopa solution (Sigma D-9628; 4 mg/ml of distilled water) was then added to each well. The reaction was allowed to proceed for 2h 47min at 30°C in a microplate reader (Thermo scientific Multiskan™ FC Microplate Photometer). Enzyme activity was defined as the slope of the reaction curve during the linear phase of the reaction (Vmax value: change in absorbance units/min) and measured using the R-based program PO-CALC [40]. All immune measurements were done blind regarding the early presence of the mother and the early exposure to pathogens.

Because the volume of extracted hemolymph and the resulting concentration of hemocytes slightly change between individuals, we standardized the concentration of hemocytes and total-PO activity (immune parameters) per microliter of hemolymph using the following formula: I x [(Vh + Vb)/Vh]/Vm, in which I is the measured immune parameter, Vh is the volume of extracted hemolymph, Vb is the volume of buffer added (i.e. 11 μl for nymphs or 25 μl for adults) and Vm is the volume applied either to the Haemocymeter for hemocyte count (i.e. 10μl) or on the spectrophotometer plate for total-PO measurement (i.e. 5μl).

### Statistical analyses

All statistical analyses were conducted using the software R v3.1.2 loaded with the packages *car, lme4, MASS* and *lsmeans*. The survival rate in between each developmental stage (defined here as “age”) of offspring (entered using the *cbind* function) was tested using a generalized linear mixed-effects model (GLMM, with binomial error distribution). In this model, the age (second, third and fourth nymphal instars, and adults), early pathogen exposure (presence/absence) and early maternal presence (presence/absence) were entered as explanatory categorical factors, while the clutch identification (ID) was entered as a random factor to control for the fact that each clutch was used for each age. Because we interested in the survival rate of nymphs until their reach adulthood, adult males and females were pooled as “adults” in this model.

Immune parameters were then analyzed separately for nymphs (for which the sex was unknown) and adults (for which the sex was known). For each nymph and adult data set, hemocyte concentration and total-PO activity were analyzed using two LMMs, in which either the age of the nymphs (second, third and fourth nymphal instars) or the gender of the adults (male or female), early pathogen exposure, early maternal presence and the weight of the measured individual were entered as explanatory factors, whereas the ID was used as a random effect. In nymphs, the weight of each class of age was scaled and centered to correct for the inherent difference in weight between each instar. All models first included all interactions between the explanatory factors and were then simplified stepwise by removing the non-significant interaction terms (all *P*-values > 0.08). Note that some non-significant interactions are presented here to allow direct comparisons between models, but their removal did not qualitatively change the results.

## Results

**Fig. 1:**
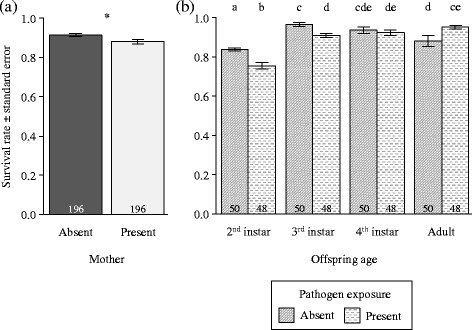
Effects of maternal presence, offspring age and pathogen exposure on offspring survival rate. The fitted values are given in function of (**a**) maternal presence and of (**b**) the interaction between pathogen exposure and offspring age in between each instar. Sample sizes are provided at the bottom of each bar. Different letters indicate statistically significant differences (*p* < 0.05). * *p* < 0.01

The presence of a tending mother overall reduced the proportion of offspring that successfully reached adulthood, independent of pathogen exposure and age (Table 1; Fig. 1a). By contrast, offspring survival depended on an interaction between pathogen exposure and age (Table 1; Fig. 1b): the pathogenic fungus *M. brunneum* reduced the survival rate of nymphs to the 2nd and 3rd instar, but did not affect their survival rate between the 3rd and 4th instars and was finally associated with an increased survival rate between the 4th instar and adulthood (Fig. 1b).

**Table 1:** Effects of age, maternal presence and pathogen exposure on the survival rate of offspring

	Survival	
	Chisq	*p*-value
Age	86.39	**<0.0001**
Maternal presence	4.00	**0.045**
Pathogen exposure	6.21	**0.013**
Age: Pathogen exposure	14.87	**0.002**

Significant *p-values* are in bold. Note that non-significant interactions are not reported in this table

**Fig. 2:**
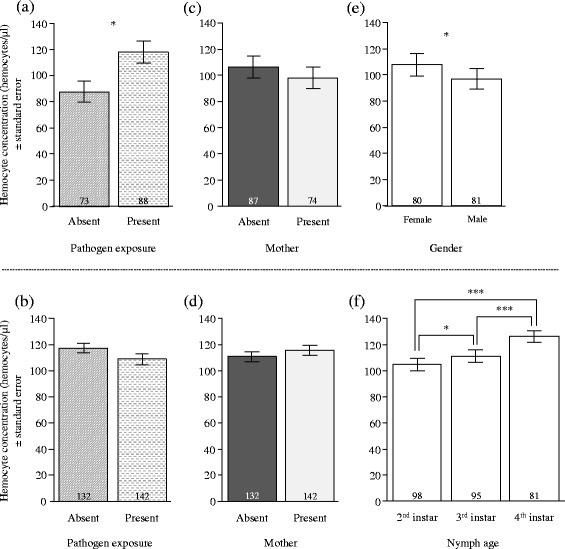
Effects of pathogen exposure, maternal presence and adult gender or nymphal age on hemocyte concentration. The fitted values are given in function of (**a**, **b**) pathogen exposure, (**c**, **d**) maternal presence and (**e**, **f**) gender/nymph age in adults and nymphs, respectively. White bars represent age/gender, grey hatched/horizontal dotted bars represent the absence/presence of pathogen, and dark/light grey bars represent the absence/presence of mother. Sample sizes are provided at the bottom of each bar. *** *p* < 0.0001

**Fig. 3:**
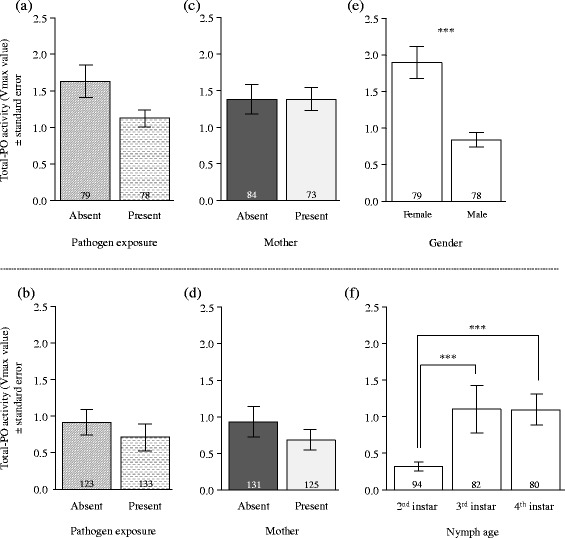
Effects of pathogen exposure, maternal presence and adult gender or nymphal age on total-PO activity. The fitted values are given in function of (**a**, **b**) pathogen exposure, (**c**, **d**) maternal presence and (**e**, **f**) gender/nymph age in adults and nymphs, respectively. Sample sizes are provided at the bottom of each bar. *** *p* < 0.0001

**Fig. 4:**
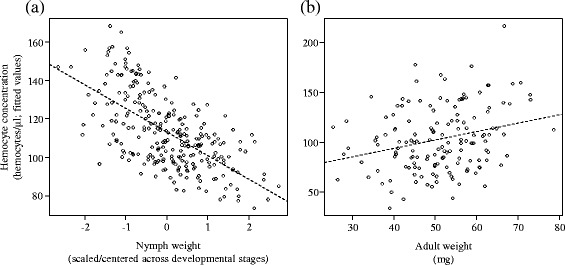
Correlation between hemocyte concentration and the weight in (**a**) nymphs and (**b**) adults. The hemocyte concentration are fitted values obtained from the LMMs. In nymphs, the weight of each class of age was scaled and centered to correct for the inherent difference in weight between each instar. Each dot represents an individual and the dash line represents correlation between hemocyte concentration and weight

Overall, there were contrasting effects of pathogen exposure, maternal presence, body weight, offspring developmental stage and adult gender on hemocyte concentration and total-PO activity in offspring. Specifically, early pathogen exposure increased hemocyte concentration, but not total-PO activity in adults (Table 2; Figs. 2a and 3a), whereas it did not affect these two immune parameters in nymphs (Table 3; Figs. 2b and 3b). Early maternal presence also had no effect on the concentration of hemocytes and on the total-PO activity in both nymphs and adults (Tables 2 and 3; Figs. 2c, d and 3c and d). By contrast, the association between body weight and hemocyte concentration was positive in nymphs (Table 3; Fig. 4a; ρ = -0.27; C.I. 95% = [−0.38;−0.16]), but negative in adults (Table 2; Fig. 4b; ρ = 0.27; C.I. 95% = [0.11; 0.41]). There was, however, no association between body weight and total-PO activity in nymphs and adults (Tables 2 and 3). Finally, the concentration of hemocytes and the total-PO activity increased between each nymphal instar (Table 3; Figs. 2f and 3f) and were higher in adult females compared to adult males (Table 2; Figs. 2e and 3e).

**Table 2:** Effects of gender, maternal presence, pathogen exposure and weight on immune parameters in adults

	Hemocyte concentration		Total-PO activity	
	Chisq	*p*-value	Chisq	*p*-value
Gender	3.97	**0.046**	42.12	**<0.0001**
Maternal presence	0.36	0.547	1.18	0.278
Pathogen exposure	4.99	**0.025**	2.25	0.133
Weight	7.38	**0.006**	0.02	0.900

Significant *p-values* are in bold. Note that non-significant interactions are not reported in this table

**Table 3:** Effects of age, maternal presence, pathogen exposure and weight on immune parameters in nymphs

	Hemocyte concentration		Total-PO activity	
	Chisq	*p*-value	Chisq	*p*-value
Age	12.65	**0.001**	74.47	**<0.0001**
Maternal presence	0.41	0.520	0.12	0.732
Pathogen exposure	0.17	0.681	0.61	0.436
Weight	18.71	**<0.0001**	0.13	0.721

Significant *p-values* are in bold. Note that non-significant interactions are not reported in this table

## Discussion

This study aimed at elucidating the effects of early maternal presence and early exposure to pathogens on the immunity of growing offspring in the European earwig *F. auricularia*. Our results show that the presence of the mother during the first 2 weeks of life has no effect on the immunity of her offspring at both nymphal and adult stages. However, maternal presence generally reduced the survival of their offspring, a result in line with a previous study [38] and suggesting that the benefits of post-hatching care in *F. auricularia* could generally take a form that is excluded from the current experimental setup (e.g. predator defense, thermal resistance). Conversely, we found that early pathogen exposure generally increased the concentration of hemocytes—but not the total-PO activity—in adult offspring and had no effect on nymph’s immunity. Both hemocyte concentration and total-PO activities increased with offspring development, and these two immune parameters were higher in adult females compared to adult males. Finally, pathogen exposure early in life caused low survival during the early developmental stages, while it increases adult survival.

Somewhat surprisingly, we found that maternal care has no long-lasting effects on offspring immunity. This result contrasts with other studies investigating short- or long- term effects of parental care/parental deprivation on offspring immunity in vertebrates [17–19, 41]. For instance, nestling immunocompetence increased with parental care 24 h after an immune challenge in barn swallows *Hirundo rustica* [17], and a long-term decrease in immunity has been shown in adult mice early-deprived of their mother [18]. The limited effects of maternal presence on offspring immunity reported here therefore reveal that maternal presence does not necessarily shape the immunity of offspring in insects, and more generally that pathogens are not a universal selective pressure promoting maintenance of post-hatching maternal care in nature. Understanding whether this independence between parental care and offspring immunity is unique to earwig biology [38, 42, 43] (e.g. juveniles are less dependent on maternal care in earwigs compared to most vertebrate and invertebrate species) or on the immune system of invertebrates in general [44] will require further studies exploring the expression and nature of this link across a larger set of species.

Early exposure to *M. brunneum* did not unmask the effect of maternal care on offspring immunity, which contrasts with a previous result demonstrating that maternal presence improves the survival of eggs exposed to fungal spores in this species [25]. However, we found an age-specific effect of pathogen exposure on offspring survival, which reflected a reduced survival rate between hatching and the 3rd instar, an absence of effect between 3rd and 4th instar and a higher survival rate between 4th instar and adulthood. This contrasting age-specific effect of pathogen exposure might reflect three non-mutually exclusive processes. First, juveniles could exhibit a weak immune activity (as reported in many vertebrate and invertebrate species, see for instance in reptiles [45] and in the honey bee [46]), making them less likely to survive pathogen exposure compared to older nymphs [3]. In line with this scenario, we found that the levels of total-PO and hemocyte concentrations increased with the developmental stage of the nymphs. Second, due to our experimental design the 2nd and 3rd developmental instars were chronologically the first instars emerging after pathogen exposure, which could have resulted in a higher proportion of live spores in the environment of young compared to old offspring and thus in a decreased risk of a novel infection in later instars. Finally, the survival of adults could reflect the early elimination of the weakest individuals in pathogen compared to control treatments, which entailed the production of better quality adults in the former case. To disentangle these three processes, further studies should thus investigate whether offspring exposed to a pathogen either at the beginning of each instar or only at their 1st instar exhibit different or similar survival rates and levels of immunity.

Besides the general increase of offspring’s immunity over developmental stages, we found that the association between hemocyte concentration and individual weight was negative in nymphs, but positive in adults. Immunity is generally sustained by either increasing the acquisition of food resources or by reducing energy allocation to other physiological processes such as growth and reproduction [2, 47] (see for examples [17, 47–49]). However, variation in the amount of resources available to an individual is known to possibly mask investment trade-offs between mutually exclusive functions and even to produce positive associations between these functions at a population level [42, 50, 51]. The apparent discrepancy between the presence of a trade-off in nymphs and of a positive association in adults therefore suggests that investing into immunity is generally costly in earwigs, but that this cost is masked in adults—possibly due to a higher variation in resource acquisition between adults compared to between nymphs. Investigating variation in foraging strategies and food intake of nymph and adult earwigs, and thus their role in immune investment will be done in the future. This notwithstanding, our results also reveal that maternal presence does not limit the costs of immune investment, further stressing the limited effects of maternal care on offspring immunity.

Independent of body weight, our results finally showed that adult females had more hemocytes and a higher total-PO activity than adult males. This sex-specific investment into immune components is in line with results found across many insect species, such as butterflies [52], crickets [53], dragonflies [54], flies [55] and scorpionflies [56]. In earwigs, males are known to survive only for a single reproductive period (i.e. a few months). By contrast, females live up to 1.5 years, during which they provide care to their eggs for several months, provide care to the resulting nymphs for several weeks and then often produce a second clutch which they care for during several additional weeks [32, 37]. Compared to males, females fitness is therefore tightly associated with their capability to survive over several seasons and thus to fight against longer and/or more frequent attacks by pathogens, overall likely explaining their higher investment into immune defense (see also [33]).

## Conclusions

Our results overall reveal that age, gender and parasite exposure shape the immune system of the European earwig *F. auricularia*, while the presence of a caring mother did not. Personal immunity and social immunity in the form of maternal care are nevertheless not the only protection against pathogens that can operate within family units [16]. For instance, larvae can participate in social immunity and thus provide immune benefits to their siblings by sanitizing the nest with anal exudates, a phenomenon reported in the burying beetle *N. vespilloides* [57] and importantly, in the European earwig *F. auricularia* [58]. Our findings therefore reveal that for nymphs, the net benefits of family interactions in terms of protection against pathogen infection are unlikely to come from the mothers, but could instead result from the presence and/or interactions with their siblings. Hence, our findings overall call for further studies investigating the role of sibling behaviors, together with age, gender and parasite exposure, in the emergence and maintenance of family life in nature.
